# Outbreaks of Root Rot Disease in Different Aged American Ginseng Plants Are Associated With Field Microbial Dynamics

**DOI:** 10.3389/fmicb.2021.676880

**Published:** 2021-06-25

**Authors:** Li Ji, Fahad Nasir, Lei Tian, Jingjing Chang, Yu Sun, Jianfeng Zhang, Xiujun Li, Chunjie Tian

**Affiliations:** ^1^Key Laboratory of Mollisols Agroecology, Northeast Institute of Geography and Agroecology, Chinese Academy of Sciences, Changchun, China; ^2^University of Chinese Academy of Sciences, Beijing, China; ^3^School of Life Sciences, Jilin Agricultural University, Jilin, China

**Keywords:** American ginseng, disease outbreaks, microbial communities, soil physicochemical properties, pathogens

## Abstract

American ginseng (*Panax quinquefolium* L.) is a perennial plant that is cultivated for medicinal purposes. Unfortunately, outbreaks of root rot disease in American ginseng (AG) reduce yields and result in serious economic losses. Information on the dynamics of soil microbial communities associated with healthy and diseased AG of different ages is limited. The present study explored the differences in field soil microbial community structure, composition, interaction, and their predictive functions associated with healthy and diseased AG at different growth ages. Changes in soil physicochemical properties were also examined to determine the possible reasons for disease outbreaks. Results revealed that in different growth years, the genera of soil-borne pathogens, such as *Alternaria*, *Botrytis*, *Cladosporium*, *Sarocladium*, and *Fusarium*, were increased in diseased AG soil samples in comparison with those in the healthy AG soil samples. In contrast, the abundance of some key and potentially beneficial microbes, such as *Bacillus*, *Chaetomium*, *Dyella*, *Kaistobacter*, *Paenibacillus*, *Penicillium*, and *Trichoderma*, was decreased. Additionally, as AG plants age, the relative abundance of symbiotic fungi tended to decrease, while the relative abundance of potential plant pathogenic fungi gradually increased. Various soil properties, such as available phosphorus, the ratio of total nitrogen to total phosphorus (N/P), and pH, were significantly (*P* < 0.05) associated with microbial community composition. Our findings provide a scientific basis for understanding the relationship among the root rot disease outbreaks in American ginseng as well as their corresponding soil microbial communities and soil physicochemical properties.

## Introduction

Medicinal plants are commonly used in treating and preventing specific diseases and are considered as the foundation of traditional Chinese medicine. As a result, they have been widely recognized to play a beneficial role in healthcare (Singh, 2015; Shi et al., 2018). American ginseng (*Panax quinquefolium* L.), a perennial herbaceous plant (Zou et al., 2015), is highly sought for its medicinal value and represents one of the best-selling medicinal herbs in the world (Christensen, 2009; Lui et al., 2012). In recent years, with the increased research on the pharmacological activities of American ginseng (AG) and the rapid development of the Chinese medicine industry (Normile, 2003), the demand for AG has been increasing (Qi et al., 2010). Due to its slow growth property, it typically takes 4 years of growth for AG to reach optimal growth size and ginsenoside content (Farh et al., 2018). Unfortunately, the biggest limitation of AG in cultivation management is that it is extremely susceptible to soil-borne diseases (Punja, 2011; Kochan et al., 2013). Root rot is among the soil-borne diseases, which seriously constrains the quality and yield of AG and poses a significant threat to continuous cropping of AG (Rahman and Punja, 2005; Jiang et al., 2019).

Studies have shown that root rot disease of AG is mainly caused by the soil-borne pathogens, such as *Phytophthora cactorum* (Bobev et al., 2003), *Cylindrocarpon destructans* (Reeleder et al., 2002; Farh et al., 2018), *Fusarium solani*, and *F. oxysporum* (Punja et al., 2007; Bi et al., 2011). Although the specific microbes can directly or indirectly invade or protect plants, their efficacy is largely influenced by the entire microbial communities (Van Elsas et al., 2002; Raaijmakers et al., 2010; Berendsen et al., 2012). Several studies have reported that soil microbial community diversity, composition, function, and interactions all play an important role in plant health and are associated with plant soil-borne disease outbreaks (Cha et al., 2016; Niu et al., 2016; Wang et al., 2017). Thus, understanding the relationship between root rot disease outbreaks and soil microbial communities is of great significance for maintaining the balance of the soil microbial ecosystem and ensuring the suitability of plant growth (Anderson, 2003; Garbeva et al., 2004; Van Der Heijden et al., 2008).

Many studies have been conducted to understand the factors of microbial communities that are responsible for the health of AG. Specifically, the majority of the studies focused on the changes in microbial communities of healthy AG in different growth years (Dong et al., 2018; Zhang J. et al., 2020) or only evaluated the differences of microbial communities between healthy and diseased AG within a certain year (Jiang et al., 2019). However, the dynamic changes in the microbial communities of healthy and diseased plants over several continuous years have not been examined. Plants serve as the host for pathogens by providing a suitable habitat for them (Casadevall and Pirofski, 2000). The adaptability of pathogens to the host and soil environment may increase over time (Rahman and Punja, 2005); thus, with the growth of AG, the pathogen load may gradually increase. Furthermore, the relationship between the host and the microbiome and the microbial diversity could be easily affected by changes in the soil environment (Dangl and Jones, 2001; Dordas, 2009). Notably, several studies have reported that changes in microbial communities in soils in which ginseng is cultivated are closely related to edaphic factors (Punja et al., 2008; Li et al., 2018). Therefore, elucidating the soil physicochemical properties that induce changes in microbial community composition during the continuous growth of AG, especially in soils in which diseased AG is present, is essential and could provide a theoretical foundation for the prevention and control of AG diseases and soil improvement.

The main objectives of the current study were to (i) investigate the changes in soil microbial communities associated with AG root rot disease in different growth ages and (ii) explore the microbial dynamics that are related to the age of AG plants and are affected by soil physicochemical properties. To this end, we compared the physicochemical properties of soils in the root zone of healthy and diseased AG of different ages, as well as the microbial community composition, structure, and interactions in these soils. As an outcome of this study, we provided a perspective of understanding the root rot disease outbreaks of different AG growth ages in ginseng fields.

## Materials and Methods

### Sampling

In August 2019, the soil samples surrounding ginseng roots of 2-, 3-, and 4-year-old healthy (H2, H3, H4) and diseased (D2, D3, D4) AG plants were collected from a field-grown AG located in Ji’an City, Jilin Province, China (126°11′33′′E, 41°10′2′′N) (Supplementary Figure 1). This area of northeast China is one of the main production areas for the cultivation of AG under field conditions. The altitude is 250 m, the mean annual temperature is 6.5°C, the frost-free period is about 150 days, the annual precipitation is 800–1,000 mm, and the soil type is a dark brown soil. In this AG planting field, the 2-, 3-, and 4-year-old AG plants were planted in 1.8-m-wide and 20-m-long seedbeds, respectively. The distance between the seedbeds was approximately 0.5 m, the plant spacing on the seedbeds was about 10 cm, and the row spacing was about 20 cm. The health and/or serious root rot disease status of AG plants was based on observation. For instance, plants with firm stems, green leaves, and roots free of rust spots, rot, and no other disease symptoms were considered healthy plants. In contrast, yellow and wilted plants containing more than half of rotten roots were considered as materials exhibiting serious root rot disease. From the same aged AG plants, five samples with the described healthy or diseased appearance were randomly selected and pooled together as a replicate. Four replicates of each soil sample from both the healthy and diseased AG plants at different ages were collected by excavating of soil (about 20 cm) and were gently shaken to collect root zone soil. All soil samples (2 healthy states × 3 growth years × 4 replicates, total 24 samples) were packed in zip-locked bags and divided into two subsamples. One subsample was air dried at room temperature for soil physiochemical analysis, and another portion was stored at −80°C for DNA extraction and subsequent sequence analysis.

### Soil Physicochemical Analysis

Soil pH and electrical conductivity (EC) were measured following the methods of Ji et al. (2021). Soil organic matter (SOM), total nitrogen (TN), total phosphorus (TP), and KMnO_4_-oxidizable carbon (KMnO_4_-C) were evaluated as previously described (Luo et al., 2017). Soil available phosphorus (AP) and available potassium (AK) were evaluated using the methods described in the previous study of Shi et al. (2019).

### DNA Extraction and Amplification Sequencing

DNA extraction, concentration detection, and purification were as previously described (Ji et al., 2021). The V3–V4 hypervariable regions of the bacterial *16S rRNA* gene were amplified using the primer pair of 341F (5′-CCTACGGGNGGCWGCAG-3′) and 806R (5′-GGACTACHVGGGTWTCTAAT-3′). The fungal ITS1 region was amplified using the primers ITS 5F (5′-GGAAGTAAAAGTCGTAACAAGG-3′) and ITS 1R (5′-GCTGCGTTCTTCATCGATGC-3′) (Kõljalg et al., 2005; Chang et al., 2019). The 250-bp paired-end sequencing of the PCR amplicons was performed using an Illumina MiSeq PE250 platform (Biomarker Technologies Co., Ltd., Beijing, China). The raw sequences were processed using QIIME (v2.0) and merged by FLASH (v1.2.7) to obtain raw tags (Magoč and Salzberg, 2011), and the raw tags were filtered using Trimmomatic (v0.33) (Bolger et al., 2014). UCHIME (v4.2) was used to remove chimeric sequences and obtain a final set of clean tags (Edgar et al., 2011). UCLUST in QIIME (v2.0) was used to group the amplicon sequences into operational taxonomic units (OTUs) at a 97% sequence similarity. OTU taxonomy classification was assigned to each phylotype using the RDP (v2.2) classifier and the Greengenes database (gg_13_8) for bacteria and the UNITE database (v 7.1) for fungi (DeSantis et al., 2006; Abarenkov et al., 2010). The fungal OTUs were assigned ecological guilds using FUNGuild (v1.0) (Nguyen et al., 2016).

### Bioinformatics and Statistical Analysis

Alpha diversity indices, including Chao1 richness and Shannon diversity, were performed using the ‘‘phyloseq’’ package in Microbiome Analyst^1^ (Dhariwal et al., 2017). The “Venn diagram” package in R (v3.6.1) was used to visualize the numbers of shared and unique OTUs between different samples. Between-class analysis (BCA) and co-inertia analysis were used to explore the variation in the structure of the bacterial and fungal communities in soil samples by the “ade4” package in R (v3.6.1) (Abe et al., 2007). The abundance of potential symbiotic and pathogenic fungal species was assessed using the FUNGuild online website^2^ (Nguyen et al., 2016). The linear discriminant analysis (LDA) effect size (LEfSe) method was used to determine the biomarkers in different samples, using the non-parametric Kruskal–Wallis rank sum test (Segata et al., 2011). Co-occurrence analysis was conducted at the genus level using the pairwise Spearman’s rank correlation coefficients (Spearman’s | RHO| > 0.9, *P* < 0.01), while Gephi (v0.9.2) was used to calculate the network topology characteristics. In addition, nodes and links were calculated based upon the robustness of the co-occurrence scores (Bastian et al., 2009). NetShift analysis was used to visualize the potential keystone driver taxa based on differences in network interactions between healthy and diseased plants (Kuntal et al., 2019)^3^. Redundancy analysis (RDA) was performed by “vegan” packages in R (v3.6.1) (Jiang et al., 2019) to determine if changes in the microbial communities were related to the soil environment.

The number of OTUs, alpha diversity indices, and relative abundance of bacteria and fungi were calculated for all replicates and subjected to analysis of variance by one-way ANOVA (*P* < 0.05). A Welch’s *t*-test and one-way analysis of variance (Duncan’s multiple test) were used to compare mean differences between samples using SPSS (v20.0). All data with differences at *P* < 0.05 were considered statistically significant.

### Data Availability

The bacterial and fungal raw sequence data have been submitted to the National Center for Biotechnology Information (NCBI) and can be accessed using accession numbers PRJNA634905 and PRJNA634903.

## Results

### Illumina MiSeq Sequencing Data

A total of 4,754,490 *16S rRNA* clean tags were obtained from the 24 samples (ranging from 68,768 to 227,264), with an average of 165,223 clean tags per sample, producing a total of 5,821 OTUs at a 97% similarity level. In addition, 3,498,738 ITS reads (ranging from 95,290 to 146,744) were obtained from the 24 samples, which generated 624 OTUs at a 97% similarity level. The rarefaction curves tended to approach the saturation plateau, indicating that the sequencing depth was sufficient enough to detect most of the generated amplicon sequences (Supplementary Figure 2).

### Soil Bacterial and Fungal Community Diversity

**FIGURE 1:**
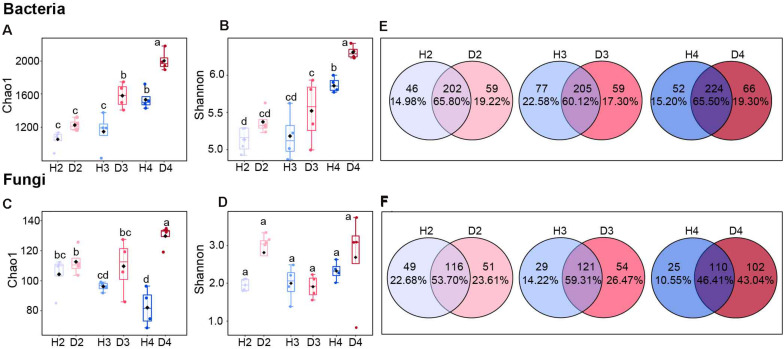
Chao1 and Shannon indices of bacterial **(A,B)** and fungal **(C,D)** communities. Different letters above the bars indicate significant differences among samples. Significant differences between sample groups were determined by one-way ANOVA followed by Duncan’s multiple range test, *P* < 0.05; Venn diagrams of bacteria **(E)** and fungi **(F)** at the genus level in soils obtained from different aged healthy and diseased AG plants. H2, soils obtained from 2-year-old healthy AG plants; D2, soils obtained from 2-year-old diseased AG plants; H3, soils obtained from 3-year-old healthy AG plants; D3, soils obtained from 3-year-old diseased AG plants; H4, soils obtained from 4-year-old healthy AG plants; D4, soils obtained from 4-year-old diseased AG plants.

The microbial alpha diversity in soil samples derived at different age stages of healthy and diseased AG plants indicated that the bacterial Chao1 and Shannon indices tended to increase with increasing plant age. The bacterial Chao1 and Shannon indices of H4 samples were significantly higher than H2 and H3 samples, but significantly lower than the D4 samples (Figures 1A,B). The Chao1 index for fungi in the healthy ginseng samples exhibited a decreasing trend with increasing plant age (Figure 1C), while no significant difference was observed in the Shannon index (Figure 1D). The Venn diagram also illustrates that the proportion of unique fungal groups in healthy samples of different aged plants was lower than that in diseased samples of different aged plants, where the proportion of unique fungal groups increased with increasing plant age (Figures 1E,F).

**FIGURE 2:**
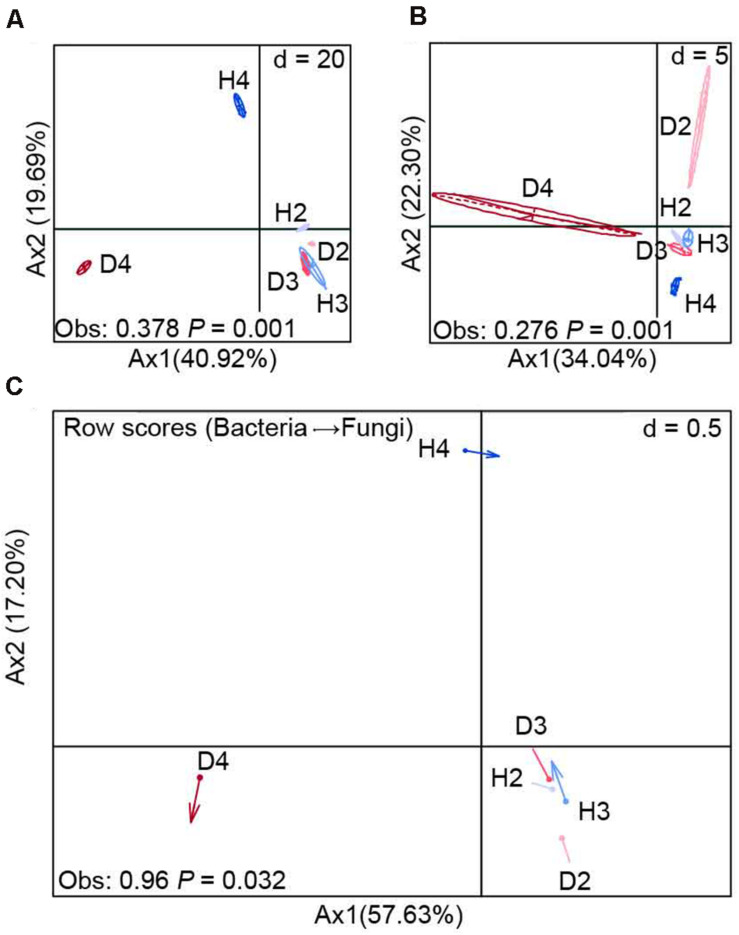
Between-class analysis of bacterial **(A)** and fungal **(B)** communities (OTU level) and their co-inertia **(C)**. H2, soils obtained from 2-year-old healthy AG plants; D2, soils obtained from 2-year-old diseased AG plants; H3, soils obtained from 3-year-old healthy AG plants; D3, soils obtained from 3-year-old diseased AG plants; H4, soils obtained from 4-year-old healthy AG plants; D4, soils obtained from 4-year-old diseased AG plants.

BCA at the OTU level revealed that there were significant (*P* = 0.001) differences in the composition of bacterial and fungal communities between the soil samples obtained from healthy and diseased AG plants, especially 4-year-old plants (Figures 2A,B). This finding was further supported by the results of the co-inertia analysis, which showed that the healthy and diseased AG plants of different growth ages explained 74.83% of the total microbial community variation (*P* = 0.032). In addition, the projection of arrows in the samples obtained from healthy and diseased AG plants pointing in different directions indicates that different growth aged AG plants had a weak similarity on the variation of bacterial–fungal communities between diseased and healthy samples (Figure 2C).

### Composition of the Soil Bacterial and Fungal Communities and Functional Predictions

**FIGURE 3:**
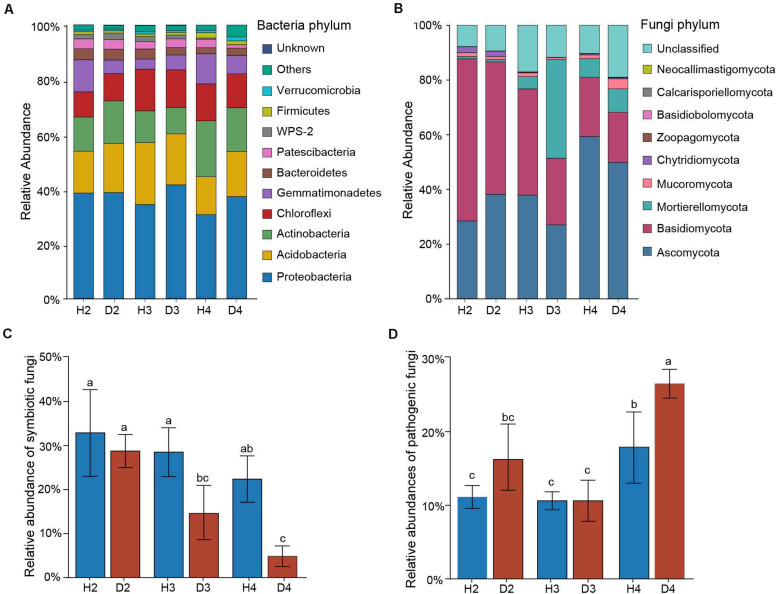
Relative abundances of the top 10 most abundant bacteria **(A)** and fungi **(B)** at the phylum level in soil samples obtained from different aged healthy and diseased AG plants. Relative abundances of symbiotic **(C)** and pathogenic **(D)** fungi detected in the soil of different aged healthy and diseased AG plants based on FUNGuild analysis. The error bars represent standard deviations of the means. Different letters above the bars indicate significant differences among samples at *P* < 0.05 based on one-way ANOVA followed by Duncan’s multiple range test. H2, soils obtained from 2-year-old healthy AG plants; D2, soils obtained from 2-year-old diseased AG plants; H3, soils obtained from 3-year-old healthy AG plants; D3, soils obtained from 3-year-old diseased AG plants; H4, soils obtained from 4-year-old healthy AG plants; D4, soils obtained from 4-year-old diseased AG plants.

Changes in the relative abundance at the phylum level in microbial communities were first assessed to identify the microbial taxa underlying the observed structural differences associated with different aged AG plants. The top five abundant bacterial phylum in all 24 soil samples were Proteobacteria (30.69–66.15%), Acidobacteria (13.83–22.64%), Actinobacteria (9.63–22.64%), Chloroflexi (9.22–15.23%), and Gemmatimonadetes (3.67–11.70%) (Figure 3A). The soil fungal community was mainly dominated by Ascomycota (27.02–59.32%), Basidiomycota (18.31–59.23%), and Mortierellomycota (0.83–8.55%) in all soil samples (Figure 3B). Since most of the AG diseases are caused by fungi, the abundances of potential symbiotic and pathogenic fungal species were further analyzed (Supplementary Table). Results indicated that the abundance of symbiotic fungi (e.g., *Amphinema*, *Lecythophora*, *Russula*, and *Wilcoxina*) tended to decrease with increasing plant age, and they were significantly lower in the healthy samples than in the diseased ones of 3- and 4-year-old AG plants (Figure 3C). In contrast, the abundance of potential plant pathogenic fungi (e.g., *Alternaria*, *Cladorrhinum*, *Leptosphaeria*, and *Strelitziana*) gradually increased with increasing plant age. Notably, the abundance of potential plant pathogens in samples obtained from both healthy and diseased 4-year-old AG plants was significantly higher than in all of the other age samples, and the relative number in D4 samples was higher than in H4 samples (Figure 3D).

**FIGURE 4:**
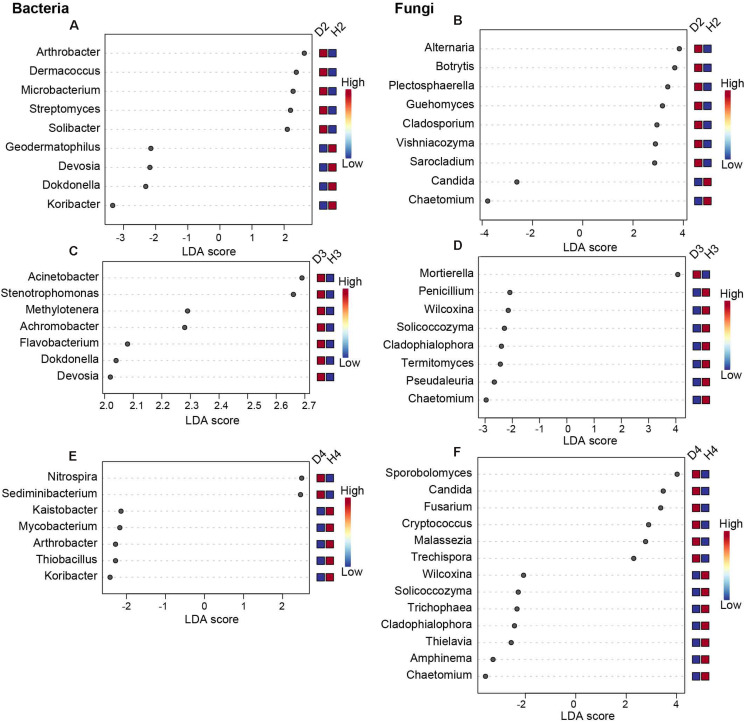
Biomarkers of healthy and diseased AG microbiota at different plant ages, calculated using the LEfSe method. Only results with | LDA| > 2, *P* < 0.05, in Tukey’s honestly significant difference test are shown. **(A,C,E)** Represent the bacterial biomarkers in soil samples obtained from 2-, 3-, and 4-year-old AG plants, respectively, **(B,D,F)**, represent the fungal biomarkers in soil samples obtained from 2-, 3-, and 4-year-old AG plants, respectively. H2, soils obtained from 2-year-old healthy AG plants; D2, soils obtained from 2-year-old diseased AG plants; H3, soils obtained from 3-year-old healthy AG plants; D3, soils obtained from 3-year-old diseased AG plants; H4, soils obtained from 4-year-old healthy AG plants; D4, soils obtained from 4-year-old diseased AG plants.

To further distinguish the different responses of the soil microorganisms of healthy and diseased AG at different growth ages, LEfSe analysis was performed (Figure 4, *P* < 0.05, LDA > 2). Results revealed 18 significant differences in the microbial genera present in soil samples obtained from 2-year-old healthy and diseased AG plants. Specifically, the genera *Arthrobacter*, *Dermacoccus*, *Microbacterium*, *Streptomyces*, *Alternaria*, *Botrytis*, *Plectosphaerella*, *Guehomyces*, *Cladosporium*, *Vishniacozyma*, and *Sarocladium* were more abundant in D2 samples than in H2 samples (Figures 4A,B). A total of 15 significant differences in microbial genera were observed in soil samples obtained from 3-year-old healthy and diseased AG plants. The genera *Acinetobacter*, *Stenotrophomonas*, *Methylotenera*, *Achromobacter*, *Flavobacterium*, *Dokdonella*, *Devosia*, and *Mortierella* were dramatically higher in D3 samples than in H3 samples (Figures 4C,D). A total of 20 significant differences were observed in the soil microbial genera present in soil samples obtained from 4-year-old healthy and diseased AG plants, including *Nitrospira*, *Sediminibacterium*, *Sporobolomyces*, *Candida*, *Fusarium*, *Cryptococcus*, *Malassezia*, and *Trechispora*, which were more abundant in D4 samples than in H4 samples (Figures 4E,F). Although different aged AG plants could recruit different microbes after being infected, it is worth noting that the relative abundance of *Chaetomium* was significantly reduced in soil samples obtained from diseased AG plants of different ages (Figure 4).

### Interactions Between the Soil Bacterial and Fungal Communities

**FIGURE 5:**
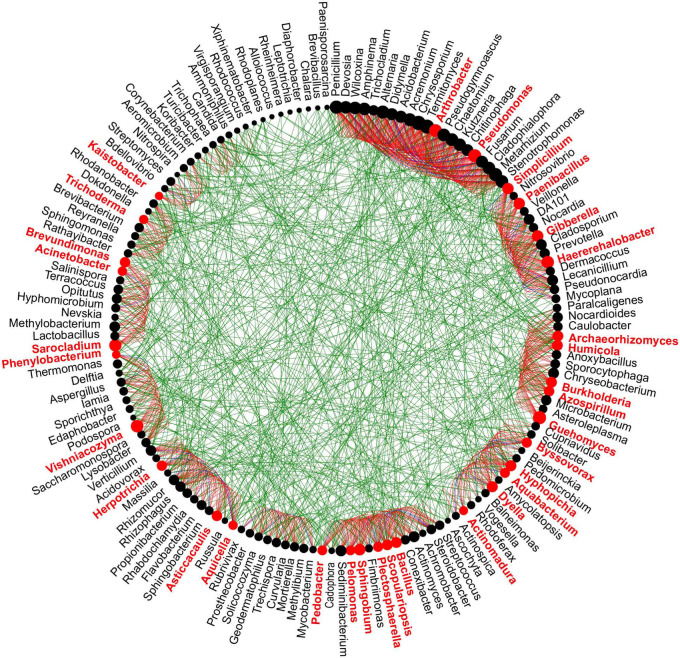
Potential “driver taxa” in the soil obtained from 2-year-old AG plants as determined in microbial co-occurrence networks between soils obtained from healthy and diseased AG plants. Node sizes are proportional to their scaled NESH (neighbor shift) score (a score identifying important microbial taxa of microbial association networks), and a node is colored red if its betweenness increases when comparing soil microbiomes associated with healthy and diseased plants. As a result, large red nodes denote particularly important driver taxa, and these taxa names are shown in red color. Green edges: association present only in healthy ginseng microbiomes; red edges: association present only in diseased plant microbiomes; blue edges: association present in both healthy and diseased plant microbiomes.

To better understand the potential interaction between bacterial and fungal genera in healthy and diseased AG plants from different age groups, microbial networks were constructed. The co-occurrence networks constructed of different aged diseased samples had a higher number of nodes, correlations, and clustering than the networks of different aged healthy samples (Supplementary Figure 3). The number of positive correlations was greater than the number of negative correlations in all samples; however, compared with H3 samples, the difference in the number of positive and negative correlations in H2 vs. H4 samples was smaller, while the number of correlations in H2 and H4 samples was lower than in H3 samples (Supplementary Figure 3B). Based on “NetShift” analysis, the driver microbes in soil samples obtained from different aged healthy and diseased AG plants were identified. A total of 32 driver genera (in red color) were identified in samples obtained from 2-year-old AG plants, including *Arthrobacter*, *Kaistobacter*, *Plectosphaerella*, *Sarocladium*, *Trichoderma*, and *Vishniacozyma*, which represent potential keystone taxa responsible for pathogen infection in the 2-year-old AG plants (Figure 5). A total of 51 genera, including *Amphinema*, *Achromobacter*, *Chaetomium*, *Didymella*, *Fusarium*, *Kaistobacter*, *Nitrospira*, *Penicillium*, *Trichoderma*, and *Wilcoxina*, were the keystone genera identified in soil samples obtained from 3-year-old AG plants (Supplementary Figure 4A). The genera *Amphinema*, *Chaetomium*, *Dyella*, *Didymella*, *Kaistobacter*, *Mycobacterium*, *Malassezia*, and others were among the 33 keystone genera identified in soil samples obtained from 4-year-old AG plants (Supplementary Figure 4B).

### Soil Physicochemical Properties and Their Relationship With Soil Microbial Community

**FIGURE 6:**
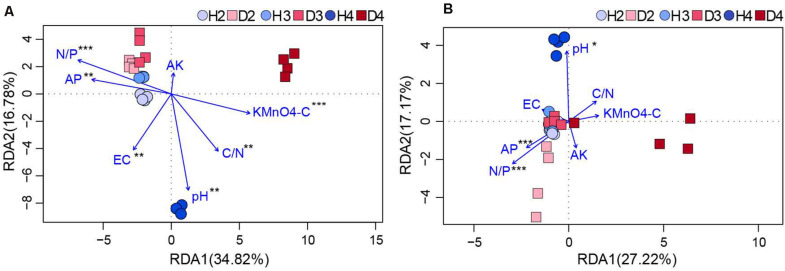
RDA of the correlation between bacterial **(A)** and fungal **(B)** communities (OTU level) with physicochemical factors of the obtained soil samples (*0.01 < *P* ≤ 0.05, **0.5 < *P* ≤ 0.01, and ***0.01 < *P* ≤ 0.001. AP, available phosphorus; AK, available potassium; KMnO_4_-C, KMnO_4_-oxidizable carbon; EC, electrical conductivity; C/N, the ratio of soil organic matter to soil total nitrogen; N/P, the ratio of soil total nitrogen to soil total phosphorus). H2, soils obtained from 2-year-old healthy AG plants; D2, soils obtained from 2-year-old diseased AG plants; H3, soils obtained from 3-year-old healthy AG plants; D3, soils obtained from 3-year-old diseased AG plants; H4, soils obtained from 4-year-old healthy AG plants; D4, soils obtained from 4-year-old diseased AG plants.

Changes in the physicochemical properties of soil samples associated with different aged healthy and diseased AG plants are shown in Table 1. The content of AP, AK, and the ratio of N to P (N/P) in H3 and H4 samples was lower than in H2 soil samples, and pH also exhibited a decreasing trend. Additionally, the pH in soil samples obtained from different aged diseased AG plants was lower than it was in soil samples obtained from healthy AG plants of different ages (Table 1). An RDA analysis was performed to further analyze the impact of soil properties on microbial communities. Results showed that the composition and structure of the bacterial community were associated with more soil properties than the fungal community (Figure 6). More specifically, soil N/P (*R*^2^ = 0.714; *P* = 0.001), KMnO_4_-C (*R*^2^ = 0.503; *P* = 0.001), AP (*R*^2^ = 0.484; *P* = 0.003), the ratio of C to N (C/N) (*R*^2^ = 0.459; *P* = 0.005), and pH (*R*^2^ = 0.409; *P* = 0.003) were all significantly correlated with the composition of the bacterial community (Figure 6A). In regard to the composition of the fungal community, a significant correlation was observed with soil N/P (*R*^2^ = 0.818; *P* = 0.001), AP (*R*^2^ = 0.432; *P* = 0.001), and pH (*R*^2^ = 0.309; *P* = 0.028) (Figure 6B). Results of RDA analysis also indicated that the soil physiochemical properties explained a higher proportion of bacterial variability (51.60%) than fungal variability (44.39%). Moreover, pH values were negatively related to most of the soil nutrients, including AP, AK, and N/P, in the analysis of both bacterial and fungal communities (Figure 6).

**TABLE 1:** Physicochemical properties of soils associated with different aged, healthy and diseased American ginseng (AG) plants.

	H2	D2	H3	D3	H4	D4
AP (mg/kg)	81.52 ± 3.99a	85.10 ± 12.40a	47.88 ± 6.83cd	64.35 ± 7.57b	52.18 ± 4.29bc	35.00 ± 6.12d
AK (mg/kg)	567.00 ± 8.98a	361.5.33 ± 19.71b	161.67 ± 1.25d	296.67 ± 1.25b	225.00 ± 13.59c	352.00 ± 17.68b
KMnO_4_-C (mg/g)	4.06 ± 0.385ab	3.68 ± 0.80b	3.23 ± 0.43b	2.32 ± 0.35c	4.13 ± 0.38ab	3.84 ± 0.62a
EC (μS/cm)	317.33 ± 7.50a	176.17 ± 10.91c	55.27 ± 6.76e	82.93 ± 2.83d	223 ± 26.15b	61.17 ± 5.60de
pH	6.43 ± 0.02a	5.79 ± 0.04d	5.94 ± 0.09c	5.77 ± 0.01d	5.76 ± 0.02d	5.49 ± 0.02e
C/N	12.56 ± 0.80a	11.68 ± 0.57ab	10.84 ± 0.44b	10.33 ± 0.72b	12.70 ± 0.69a	12.47 ± 0.54a
N/P	0.53 ± 0.02ab	0.56 ± 0.22a	0.48 ± 0.05c	0.50 ± 0.016bc	0.43 ± 0.004d	0.39 ± 0.026d

*The data represent the mean ± standard deviation (n = 4), different small letters indicate a significant (P < 0.05) difference in the measured soil property between the different soil samples as determined by a Tukey’s multiple comparison test. H2, soils obtained from 2-year-old healthy AG plants; D2, soils obtained from 2-year-old diseased AG plants; H3, soils obtained from 3-year-old healthy AG plants; D3, soils obtained from 3-year-old diseased AG plants; H4, soils obtained from 4-year-old healthy AG plants; D4, soils obtained from 4-year-old diseased AG plants; AP, available phosphorus; AK, available potassium; KMnO_4_-C, KMnO_4_-oxidizable carbon; EC, electrical conductivity; C/N, the ratio of soil organic matter to soil total nitrogen; N/P, the ratio of soil total nitrogen to soil total phosphorus.*

## Discussion

Microbial communities are regarded as an essential living component of soil, and assessing their diversity and composition can be used to evaluate soil health, which is considered to be closely related to the suppression and outbreak of soil-borne diseases (Garbeva et al., 2004; Yuan et al., 2020). Therefore, characterizing the diversity and comparison of the microbial communities in the soil surrounding healthy and diseased AG plants is a necessary step toward understanding the impact of microbial communities on the occurrence of disease in ginseng plantings (Jiang et al., 2019). Our findings revealed that soil samples obtained from diseased AG plants harbor a relatively higher bacterial and fungal Chao 1 index than soil samples obtained from healthy plants, especially in the soil samples obtained from 4-year-old AG plants (Figures 1A,C). This difference was also reflected in the number of unique fungal genera (Figure 1F). These results are in accordance with a previous study, which reported that the diseased soil samples exhibited a higher number of bacterial and fungal OTUs and Chao 1 richness compared with the disease-free soil samples (Zhou et al., 2019). Microbial diversity and balance are key for healthy plants (Yan Y. et al., 2017), and previous studies have reported that microbial diversity is related to disease resistance (Berg et al., 2017; Zhou et al., 2019). For example, Berendsen et al. (2012) reported that plants attacked by soil-borne pathogens can recruit plant beneficial microbes to their rhizosphere through the regulated secretion of root exudates. In this scenario, it is plausible that diseased AG plants may recruit specific microbes to tackle disease.

Another interesting finding is that the alpha diversity of the bacterial community exhibited an increasing trend that was age related in AG plants (Figures 1A,B). This result is similar to the results of the research of Fu et al. (2015) on kiwifruit, which showed that the diversity indices of soil microbial community in five aged kiwifruit orchard soil were significantly higher than three aged soils. Since AG is an herbaceous perennial herb, the accumulation of root exudates and the deterioration of soil properties have significant influences on the diversity of the microbial community under the accumulative effect from continuous plant growth over the course of multiple years (He et al., 2009; Doornbos et al., 2012; Eisenhauer et al., 2017; Li et al., 2018). The richness of the fungal community and the number of unique fungal groups increased with plant age in soil samples obtained from diseased AG plants (Figures 1C,F). This finding contrasts with the current thought that an increase in the population of a fungal pathogen within a soil will disrupt the inherent balance of the soil fungal community, leading to a decrease in diversity (Xiong et al., 2017). In our study, however, the number of unique fungi in soil obtained from diseased AG plants increased with plant age. Overall, our data indicate that changes in microbial alpha diversity were related to the age of AG plants.

Changes in microbial diversity are inseparable from the composition of microbial communities. There is increasing evidence that specific members of the microbiome, rather than overall microbial diversity, play an essential role in deciphering plant stress resistance (Huang et al., 2019; Xu et al., 2020). In this study, Proteobacteria and Ascomycota were the dominant bacterial and fungal phyla, respectively, found overall in AG plants, which is in agreement with a previous study reported by Jiang et al. (2019). Many members of the Proteobacteria exhibit disease-suppressing activity (Mendes et al., 2011). Unfortunately, however, the abundance of Proteobacteria decreased with plant age in soil samples obtained from healthy AG plants (Figure 3A). The community composition showed that the abundance of potential symbiotic fungi also tended to decrease with increasing plant age (Figure 3C). Conversely, the abundance of potential plant pathogenic fungi in soil samples obtained from 4-year-old AG plants was significantly higher than in samples obtained from 2- and 3-year-old AG plants (Figure 3D). Symbiotic fungi are recognized to be essential for plant nutrient uptake (Pallon et al., 2007), and pathogenic fungi can strongly influence soil biotic communities by invading plant tissues and releasing harmful secretions into the soil (Garbeva et al., 2011; De Coninck et al., 2015). Based on our results, age-dependent increased susceptibility of AG plants to disease could be explained by the gradual decrease in beneficial microbes and an increase in pathogenic microbes.

Changes in the microbial composition may determine the disease-suppressing abilities of the plants (Garbeva et al., 2004). To further reveal which soil microbes may be contributing to promote or inhibit root rot disease outbreaks in different aged AG plants, we compared the abundance of bacteria and fungi at the genus level in soil samples obtained from healthy and diseased AG plants. LEfSe analysis revealed that some genera of fungal pathogens, including *Alternaria*, *Botrytis*, *Cladosporium*, *Fusarium*, and *Sarocladium*, were more abundant in soil samples obtained from different aged, diseased AG plants (Figure 4). These fungal pathogens can commonly occur and cause serious diseases in various crops (Ayyadurai et al., 2005; Kim et al., 2016) and specifically represent a serious problem in ginseng cultivation (Lu et al., 2018; Zhang X. et al., 2020). Particularly, the root rot disease caused by *Fusarium* is known as “plant cancer,” with highly aggressive disease in AG plants grown in China (Bi et al., 2011; Jiao et al., 2011). Dong et al. (2016) reported that the relative abundance of *Fusarium* is positively correlated with the mortality rate of *Panax notoginseng*. *Cladosporium*, *Fusarium*, *Sarocladium*, and other pathogens are also responsible for causing serious disease in AG plantings and were more highly abundant in soil samples derived from diseased AG plants (Figure 4). Collectively, these taxa of microorganisms may be considered as indicators of plant disease in different aged AG plants and can be used to monitor both plant and soil health.

In addition to fungal microorganisms, we also found that some genera of potentially beneficial genera, such as *Chaetomium*, *Devosia*, *Kaistobacter*, *Penicillium*, *Amphinema*, *Wilcoxina*, and others, were differentially abundant in soil samples obtained from healthy vs. diseased AG plants (Figure 4). The antifungal activity of *Chaetomium* species has been reported in potato (*Solanum tuberosum* L.) plants against the soil phytopathogenic fungi *Rhizoctonia solani* and *Fusarium oxysporum* (Tomilova and Shternshis, 2006). *Devosia*, a genus within the family Rhizobiaceae, is often used to degrade mycotoxins, such as deoxynivalenol, which is produced by pathogenic species of *Fusarium* (Rohweder et al., 2013). Species of *Kaistobacter* are considered as having significant potential for disease suppression, as they can produce a hormone that stimulates plant growth (Tsavkelova et al., 2007). Species of *Penicillium* can produce antifungal toxins that kill, inhibit growth, and replace competing fungi (Chen et al., 2019). The genera *Amphinema* and *Wilcoxina* are ectomycorrhizal fungi that function in improving the transfer and uptake of nutrients and water to their host plants and, in return, receive photosynthetic carbon from their host plants (Miyamoto et al., 2021). Overall, these microbial taxa were more abundant in the soil sample surrounding healthy AG plants, providing evidence to suggest that they may help in disease protection. Therefore, additional studies are warranted and necessary to further study the role of these microbes in controlling soil-borne diseases associated with AG plants.

Additionally, some beneficial microbes play a unique and vital role on microbiomes in community interactions (Kuntal et al., 2019; Yuan et al., 2020). The NetShift analysis constructed in our study revealed that the genera *Amphinema*, *Bacillus*, *Chaetomium*, *Dyella*, *Kaistobacter*, *Paenibacillus*, *Penicillium*, *Trichoderma*, *Wilcoxina*, and others were acting as keystone genera (Figure 5 and Supplementary Figure 4), although some of these genera did not dominate in abundance. Previous studies have also shown that these microbes are involved in pathogen suppression (Harman et al., 2004; Shanthiyaa et al., 2013). For example, some populations of *Paenibacillus* spp. and *Bacillus* spp. can inhibit the growth of plant pathogens by producing antibiotic metabolites, while others may directly induce the defense response of the host plant prior to infection (Gardener, 2004). Some species of *Dyella* can produce β-glucosidase (An et al., 2005). Notably, β-glucosidases functioning as bioactivating components in plant defense and the high expression of genes encoding β-glucosidases in pearl millet (*Pennisetum glaucum*) were reported to enhance tolerance to pathogenic fungi (Okennedy et al., 2011). The above findings collectively indicate that, in addition to microbial abundance, community interactions among microbes also serve as important indicators of disease susceptibility in AG plantings.

Plants recruit root microbes mainly from the soils where they grow (Dastogeer et al., 2020). Changes in the soil properties could result in the differences in the composition of plant-associated microbial communities (Punja et al., 2008). Based on the results of RDA analysis, our findings provided evidence that soil AP, N/P, and pH were highly correlated with the composition of soil microbial communities (Figure 6). Soil pH was also lower in diseased AG soil samples than it was in soil samples obtained from healthy AG plants, and the pH of soil associated with healthy AG plants also decreased with increasing plant age (Table 1). These results are in agreement with previous studies demonstrating that the level of soil-borne diseases is inversely proportional to soil pH (Qi et al., 2019) and that soil pH decreases in AG plantings with increasing plant age (Liu et al., 2020). Previous studies revealed that soil factors are a strong influence on microbial communities, and conversely, microbial communities shape soil properties (Chaparro et al., 2012; Sun et al., 2018). Several studies have shown that colonization of *Fusarium* can promote the secretion of phenolic acids (Gauthier et al., 2016; Yuan et al., 2018). Some phenolic acids can inhibit the formation of pathogenic microbial communities (An and Mou, 2011), while others are capable of inducing the proliferation of pathogenic microbes (Wu et al., 2016). Notably, as previously mentioned, *Fusarium* spp. is a biomarker in the soil collected from diseased AG plants (Figure 4F). The association of a decrease in soil pH with the accumulation of pathogens needs further investigation. Soil nitrogen (N) and phosphorus (P) are essential nutrients for plant growth and can influence the resistance of plants to pathogens in sustainable agricultural systems (Dordas, 2009; Datta et al., 2015). The ratio of nitrogen to phosphorus (N/P) has been used to detect plant nutrient limitations (Yan Z. et al., 2017). In summary, our results indicated that soil properties (e.g., pH, AP, and N/P) could monitor the microbial communities of AG soil and react to changes.

## Conclusion

In conclusion, this study explored the features of microbial community diversity, composition, and their interactions in soil samples obtained from different aged healthy and diseased AG plants, as well as the changes in soil physicochemical properties. Our results indicate that (i) there were significant differences in the microbial community composition and soil characteristics in the soil attached to healthy AG plants compared with that of diseased AG plants; (ii) the soil physicochemical properties and soil microbial communities in diversity, composition, and interactions shifted with the growth of AG plants; (iii) the dynamics of soil microbial communities were mainly related to the changes of soil available phosphorus (AP), the ratio of total nitrogen to total phosphorus (N/P), and pH. Our findings are expected to provide novel strategies for cropping and disease control management in AG plantings.

## Data Availability Statement

The datasets presented in this study can be found in online repositories. The names of the repository/repositories and accession number(s) can be found below: https://www.ncbi.nlm.nih.gov/, PRJNA634905; https://www.ncbi.nlm.nih.gov/, PRJNA634903.

## Author Contributions

CT conceived and designed the study. JZ and LT helped with the experiment design. LJ collected the samples, analyzed the data, and prepared the figures and tables. FN, YS, and XL helped with the improvement of the manuscript. All authors read and approved the final manuscript.

## Conflict of Interest

The authors declare that the research was conducted in the absence of any commercial or financial relationships that could be construed as a potential conflict of interest.

## Footnotes

1 https://www.microbiomeanalyst.ca/

2 http://www.funguild.org/

3 https://web.rniapps.net/netshift
