# Supplementary material for: Outbreaks of Root Rot Disease in Different Aged American Ginseng Plants Are Associated With Field Microbial Dynamics
Source: Front Microbiol. 2021 Jun 25;12:676880. doi: 10.3389/fmicb.2021.676880 (PMC8267804; doi:10.3389/fmicb.2021.676880)
Supplement: Supplementary file 2 [file Data_Sheet_2.pdf]

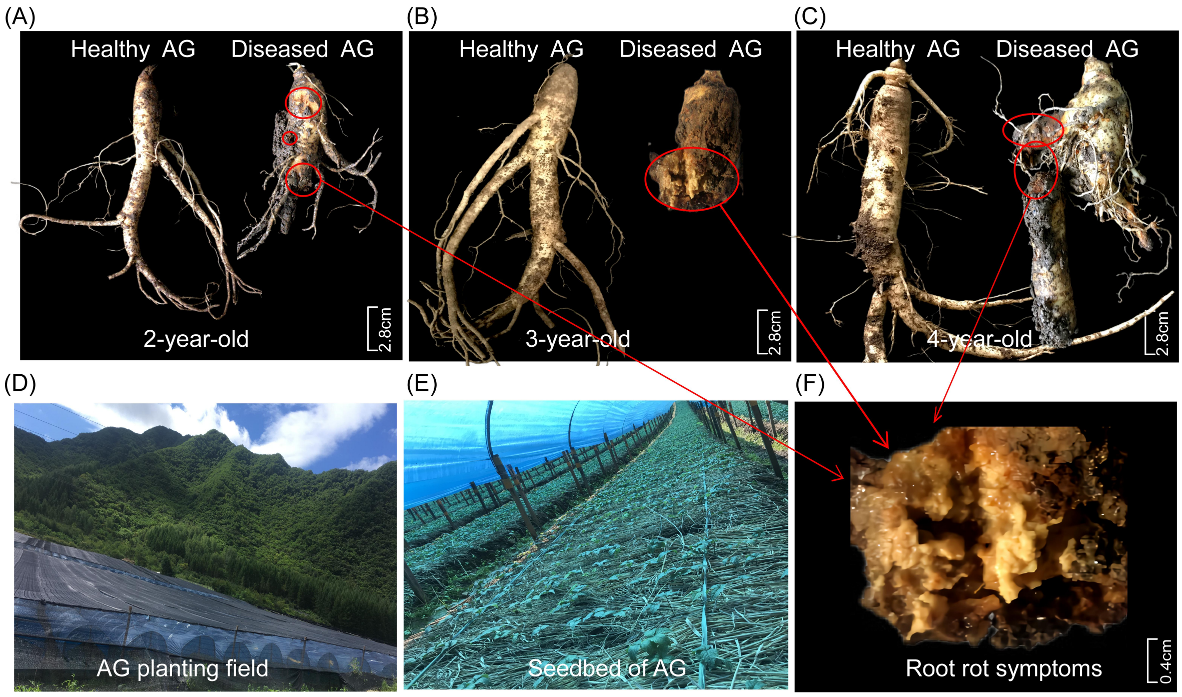

Fig. S1 Different-aged, healthy and diseased American ginseng (AG) plants collected from an AG planting field (D) and the ginseng seedbed (E). (A), (B), and (C), represent 2-year-old AG plants, 3-year-old AG plants, 4-year-old healthy AG plants, respectively. (F) represents the local symptom of root rot diseased of AG plants.

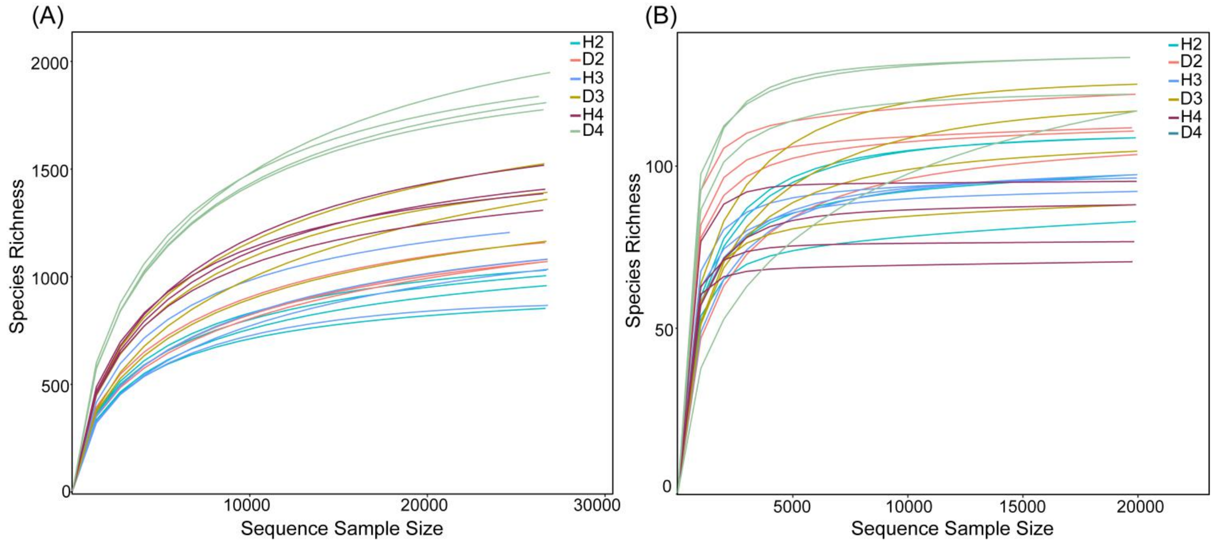

Fig. S2 Rarefaction curves of the number of OTUs plotted against the total number of sequencing reads (A: bacteria; B: fungi). H2: soil samples obtained from 2-year-old healthy AG plants; D2: soil samples obtained from 2-year-old diseased AG plants; H3: soil samples obtained from 3-year-old healthy AG plants; D3: soil samples obtained from 3-year-old diseased AG plants; H4: soil samples obtained from 4-year-old healthy AG plants; D4: soil samples obtained from 4-year-old diseased AG plants.

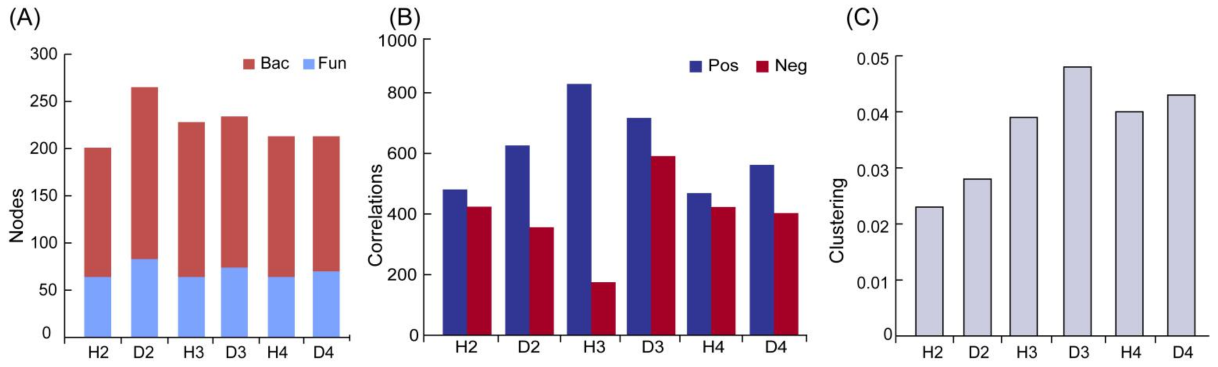

Fig. S3 Topological properties of microbial co-occurrence networks of the obtained soil samples. H2: soil samples obtained from 2-year-old healthy AG plants; D2: soil samples obtained from 2-year-old diseased AG plants; H3: soil samples obtained from 3-year-old healthy AG plants; D3: soil samples obtained from 3-year-old diseased AG plants; H4: soil samples obtained from 4-year-old healthy AG plants; D4: soil samples obtained from 4-year-old diseased AG plants.

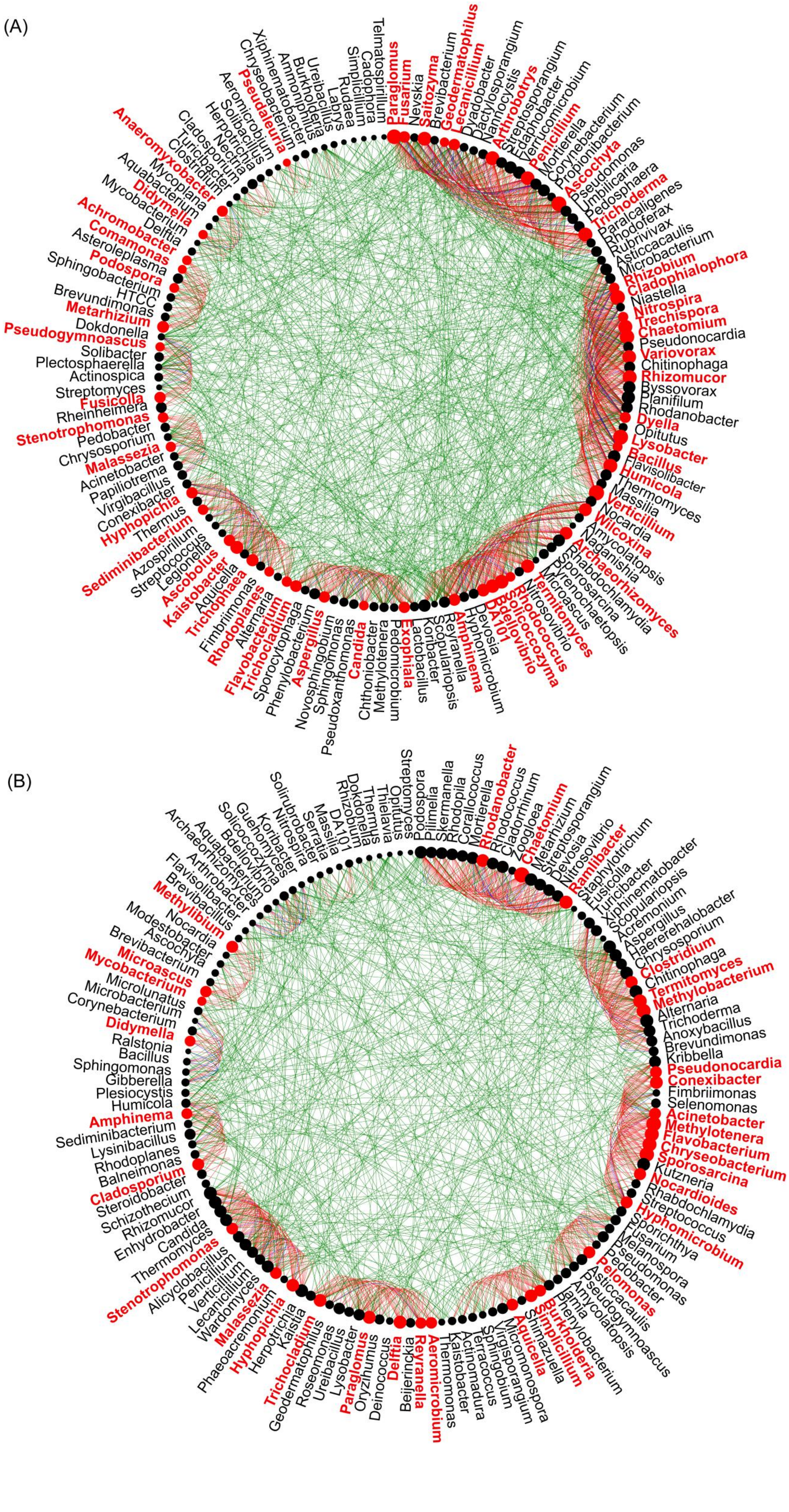

(A)
(B)

Fig. S4 Potential 'driver taxa' in soil samples obtained from 3- (A), and 4-year-old (B) AG plants based on microbial co-occurrence networks between soil samples obtained from healthy and diseased AG plants. Node sizes are proportional to their scaled NESH (neighbor shift) score (a score identifying important microbial taxa of microbial association networks), and a node is colored red if its betweenness increases when comparing soil microbiomes associated with healthy to diseased plants. As a result, large red nodes denote particularly important driver taxa and these taxa names are shown in red colour. Green edges: association present only in healthy ginseng microbiomes; red edges: association present only in diseased plant microbiomes; blue edges: association present in both healthy and diseased plant microbiomes.
